# Biodegradation of Gossypol by *Aspergillus terreus*-YJ01

**DOI:** 10.3390/microorganisms11092148

**Published:** 2023-08-24

**Authors:** Yao Jiang, Xinyue Du, Qianqian Xu, Chunhua Yin, Haiyang Zhang, Yang Liu, Xiaolu Liu, Hai Yan

**Affiliations:** School of Chemistry and Biological Engineering, University of Science and Technology Beijing, Beijing 100083, China; m202110882@xs.ustb.edu.cn (Y.J.);

**Keywords:** gossypol, *Aspergillus terreus*-YJ01, biodegradation, genomic analysis

## Abstract

Gossypol, generally found in the roots, stems, leaves, and, especially, the seeds of cotton plants, is highly toxic to animals and humans, which inhibits the use of cotton stalks as a feed resource. Here, a promising fungal strain for biodegrading gossypol was successfully isolated from the soil of cotton stalk piles in Xinjiang Province, China, and identified as *Aspergillus terreus*-YJ01 with the analysis of ITS. Initial gossypol of 250 mg·L^−1^ could be removed by 97% within 96 h by YJ01, and initial gossypol of 150 mg·L^−1^ could also be catalyzed by 98% or 99% within 36 h by the intracellular or extracellular crude enzymes of YJ01. Sucrose and sodium nitrate were found to be the optimal carbon and nitrogen sources for the growth of YJ01, and the optimal initial pH and inoculum size for the growth of YJ01 were 6.0 and 1%, respectively. To further elucidate the mechanisms underlying gossypol biodegradation by YJ01, the draft genome of YJ01 was sequenced using Illumina HiSeq, which is 31,566,870 bp in length with a GC content of 52.27% and a total of 9737 genes. Eight genes and enzymes were predicted to be involved in gossypol biodegradation. Among them, phosphoglycerate kinase, citrate synthase, and other enzymes are related to the energy supply process. With sufficient energy, β-1, 4-endo-xylanase may achieve the purpose of biodegrading gossypol. The findings of this study provide valuable insights into both the basic research and the application of *A. terreus*-YJ01 in the biodegradation of gossypol in cotton stalks.

## 1. Introduction

Protein supplies are becoming increasingly limited by animal breeding on a large scale in China [1]. Currently, China’s production of widely used soybean meal is woefully inadequate and largely dependent on imports [2]. Cottonseed meal is a reddish or yellow by-product of the cottonseed oil extraction process with a high protein content of 40%, which can be used as a high-quality plant protein feed to make up for the shortage of protein feed resources [3,4]. However, the toxic gossypol contained in cottonseed meal is as high as 0.15%~1.80%, which severely limits the use of cottonseed meal in the feed industry [5,6]. Therefore, the removal of gossypol is of great significance to the feed industry.

**Figure 1:**
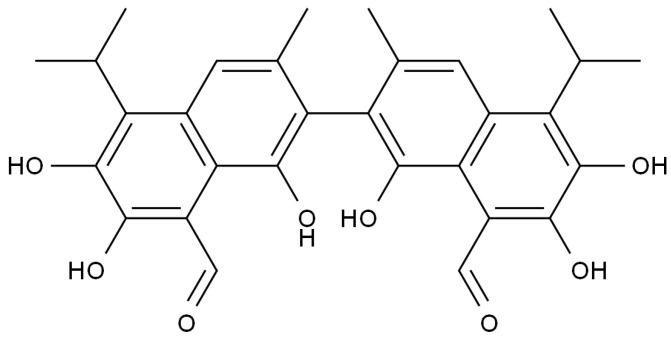
Chemical structure of gossypol (molecular weight: 518.55).

As a small molecular compound mainly derived from cottonseed, gossypol exists in two forms: bound gossypol and free gossypol (Figure 1) [7,8]. Bound gossypol is formed by combining free gossypol with proteins, amino acids, phospholipids, and other substances [9]. Due to the combination of its active groups, it loses its activity and is not absorbed by the body’s digestive system. It can be rapidly excreted in feces, usually with low toxicity [10]. Free gossypol is a major anti-nutritional factor in cottonseed meal that is detrimental to animal growth [11,12]. Gossypol is highly toxic to animals, direct feeding of gossypol can cause acute poisoning, anorexia, weakness, and even death [13,14]. In addition, gossypol can inhibit the production and activity of sperm in male animals, affect the breeding of domestic animals, and directly restrict its application in animal husbandry [15,16,17].

There are many physicochemical and biological approaches for removing gossypol and increasing the use of cottonseed products in animal feed, the most promising of which is biodegradation due to its low cost and low collateral destruction of native flora and fauna [18,19]. A number of studies have been conducted on the detoxification of gossypol, showing that microbial fermentation can significantly reduce gossypol levels in cottonseed meal [20]. The ability to biodegrade gossypol has been demonstrated in some isolated strains [21]. Some researchers studied the biodegradation of gossypol, and a few species, including *Aspergillus*, *Penicillium*, *Saccharomyces*, *Rhodococcus*, *Bacillus*, and *Lactobacillus*, were successfully isolated from the environment and found to be capable of degrading gossypol [4,22]. Hou Min et al. identified 32 strains of gossypol-tolerant intestinal endophytes by studying the similarities and differences between the intestinal microorganisms in normal and cottonseed meal diets. The highest gossypol biodegradation rate was only 90.83% [23]. Rajarathnam reported that laccase extract from rice straw colonized by a fungal mycelium (*Pleurotus florida*) had the ability to biodegrade gossypol, which led to the current study on the biodegradation of gossypol by enzymes [24]. The structure of gossypol is similar to that of naphthalene, and it can be regarded as a derivative of naphthalene [25,26]. At the same time, gossypol is a type of polycyclic aromatic hydrocarbon, and some enzymes involved in the biodegradation of polycyclic aromatic hydrocarbons have been reported, including sesquiterpene cyclase and glycosyltransferases [27,28]. Yang et al. used *Aspergillus niger* AN-1, grown on gossypol as the sole carbon source, for proteomic analysis, using MALDI-TOF MS to identify 20 protein spots involved in gossypol biodegradation, and four spots were identified as citrate synthase, kinesin family protein, and glyceraldehyde-3-phosphate dehydrogenases [29]. At present, although the biodegradation of gossypol has been widely studied, the biodegradation rate of microorganisms reported is generally low. In addition, there is no genome analysis of microorganisms for biodegrading gossypol.

In this study, an efficient fungal strain for the biodegradation of gossypol was isolated and identified as *Aspergillus terreus*-YJ01. Both YJ01 and its intracellular and extracellular crude enzymes showed effective removal of gossypol, indicating at both the cellular and enzyme level that YJ01 has a strong ability to biodegrade gossypol. Subsequently, the draft genome of YJ01 was sequenced to find genes encoding gossypol biodegradation enzymes, leading to the elucidation of the biodegradation mechanism. These findings are of great significance to the feed industry and provide an important basis for further study on the mechanism underlying gossypol biodegradation.

## 2. Materials and Methods

### 2.1. Strain and Materials

The newly isolated *A. terreus*-YJ01 used in this study was isolated from the soil of cotton straw piling in China using gossypol as the sole carbon source. Standard gossypol with a purity of 98% that was used in this study was purchased from Shanghai Ekear Biotechnology Co. All other chemicals used in the experiment were analytical grade.

### 2.2. Medium and Culture Conditions

The basal liquid medium for the isolation and culture of YJ01 consisted of 0.5 g of NH_4_Cl, 0.5 g of K_2_HPO_4_, 0.5 g of NaCl, 0.5 g of MgSO_4_, 0.1 g of peptone, 0.1 g of yeast extract powder, and 1.0 mL of trace element solution in 1000 mL of distilled water. Gossypol was added to the medium at a certain initial concentration as the sole carbon source [30], and the initial pH of the medium was adjusted to 7.0 with 1.0 mol·L^−1^ NaOH or HCl.

The Czapek Dox medium for optimal culture of YJ01 consisted of 30.0 g of sucrose, 3.0 g of NaNO_3_, 0.5 g of MgSO_4_·7H_2_O, 0.5 g of KCl, 0.01 g of FeSO_4_·4H_2_O, 1.0 g of K_2_HPO_4_, and 1000 mL of distilled water.

*A. terreus*-YJ01 was inoculated into the sterilized culture medium and grown in a 100 mL flask containing 20 mL of liquid medium. The culture condition was at 30 °C with a shaking rate of 200 r·min^−1^. Dry cell weight was measured, which represented the growth of YJ01 [31]. Both the medium and all experimental apparatuses were sterilized at 121 °C for 20 min.

A culture broth of 1.0 mL was added to a 1.5 mL centrifuge tube and centrifuged at 14,800 r·min^−1^ for 10 min. Then, 1.0 mL of acetonitrile was added to the bottom precipitation, and the gossypol in the precipitation was fully dissolved in acetonitrile using ultrasonic oscillation. The supernatant was diluted and used to determine the concentration of gossypol busing high-performance liquid chromatography (HPLC) [32,33]. The data are presented as the average values derived from three measurements, and their relative standard deviations were less than 10%.

### 2.3. Isolation and Identification of the Gossypol-Biodegrading Strain

The cotton straw pile soil sample was shaken with deionized water, and the soil suspension was inoculated into the liquid culture medium using gossypol of 500 mg·L^−1^ as the sole carbon source followed by incubation with a shake rate of 200 r·min^−1^ at 30 °C for 3 days. After three consecutive cultures, the mixed microorganisms were diluted and spread onto the solid medium containing 2% agar in the presence of 250 mg·L^−1^ gossypol. Single colonies that grew on the plates were picked and inoculated into the liquid medium containing gossypol of 250 mg·L^−1^ to test its biodegradation ability. The process was repeated several times until a pure fungal strain that had the ability to biodegrade gossypol was isolated [34].

A newly isolated strain YJ01 was identified using morphological, physiological traits, and biochemical characteristics, which was further identified using the internal transcribed spacer identification (ITS) method. The genomic DNA of YJ01 was extracted using the CTAB method using the HP Fungal DNA Kit produced by Omega Bio-tek, Inc. (Norcross, GA, USA). The purity of the extracted DNA was measured using a nanodrop 2000, and the concentration of DNA was measured using a Quantus Fluorometer (Picogreen). A polymerase chain reaction (PCR) was performed on the fungal DNA using universal primers ITS1 and ITS4. The PCR conditions were as follows: predenaturation at 95 °C for 5 min; denaturation at 95 °C for 30 s, annealing at 56 °C for 30 s, extension at 72 °C for 1 min and 30 s, 30 cycles; extension for 10 min at 72 °C. The upstream primer was 5′-TCC GTA GGT GAA CCT GCG G-3′, and the downstream primer was 5′-TCC TCC GCT TAT TGA TAT GC-3′. After the reaction, the amplified products were observed using 1% agarose gel electrophoresis, which was used to detect DNA integrity. After that, the amplified DNA was sent to Shanghai Majorbio Bio-Pharm Technology Co., Ltd. (Shanghai, China) for sequencing, and the species identification was performed using Sanger sequencing. The nucleotide sequences were compared to the most similar sequences of fungus in Gene Bank, which was used to identify this newly isolated fungal strain.

### 2.4. Preparation of the Intracellular Crude Enzyme and Extracellular Crude Enzyme

Fermentation broth of *A. terreus*-YJ01 was obtained by inoculating 1% of the preserved fungal liquid into 50 mL basal medium, which was cultured at 30 °C, 200 r·min^−1^ for 3 days. To obtain the extracellular crude enzyme, the fermentation broth was centrifuged. The filamentous fungal cells were harvested using centrifugation at 12,000 r·min^−1^ for 10 min at 4 °C, followed by washing twice with 0.01 mol·L^−1^ phosphate-buffered saline (PBS, pH 7.4). The cells were then resuspended in the phosphate buffer and sonicated at 0 °C in an ice–water mixture with an output power of 300 W for 20 min. The cell debris was removed using centrifugation at 15,000 r·min^−1^ for 10 min, and the supernatant was collected and used as an intracellular crude enzyme to catalyze gossypol [35]. The protein concentration of intracellular and extracellular crude enzymes of *A. terreus*-YJ01 was determined with the UV method. Using PBS buffer, 1.0 g·L^−1^ bovine serum protein was diluted to 0.2 g·L^−1^, 0.4 g·L^−1^, 0.6 g·L^−1^, 0.8 g·L^−1^, and 1.0 g·L^−1^, respectively. At 280 nm, the optical density of the series of concentration gradient standard solutions was measured respectively, and the standard curves for protein concentration and absorbance were drawn to calculate the protein concentration of intracellular crude enzymes [34].

### 2.5. Analysis of Gossypol Using HPLC

Gossypol content was determined using high-performance liquid chromatography (HPLC, Shimadzu LC-l0ATVP, Shimadzu Co., Kyoto, Japan) with a mobile phase of acetonitrile–0.2% phosphoric acid solution (85:15) mixture and a flow rate of 1.0 mL·min^−1^. The detection wavelength was 235 nm using a UV diode array detector coupled to an Agilent SB-C18 column (4.6 mm × 250 mm, 5 μm) at 30 °C. The calibration curve was established between the peak areas and the concentration of gossypol, which was used to calculate the concentration of gossypol in the experiment [33,36].

### 2.6. Whole Genome Sequencing of YJ01

A single colony of *A. terreus*-YJ01 was selected and inoculated into 200 mL of sterilized Czapek Dox medium. After 2 days of culture, a microscopic observation was made to ensure that the medium was not contaminated and that no mycelium formed [26,37]. The fungal cell precipitate was collected in a 1.5 mL centrifuge tube and sent to Shanghai Majorbio Bio-pharm Technology Co., Ltd. for whole genome sequencing.

Fungal genome denovo sequencing refers to sequencing the fungal genome without relying on a reference sequence and using bioinformatics tools to assemble the genome sequence from scratch [38]. The Illumina second-generation sequencing platform was used to sequence and analyze the fungal genome [39]. Qualified DNA samples were constructed with inserted fragments of 400 bp, and PE150 pair-end sequencing was performed [40]. Each sample provided original sequencing data with a coverage depth of at least 100× genome [41]. Based on the obtained sequencing data, a series of analyses were performed, including sequencing data quality control, genome evaluation, genome assembly and prediction, gene annotation, and other analyses [42,43].

## 3. Results and Discussion

### 3.1. Isolation and Identification of the Gossypol-Biodegrading Strain

**Figure 2:**
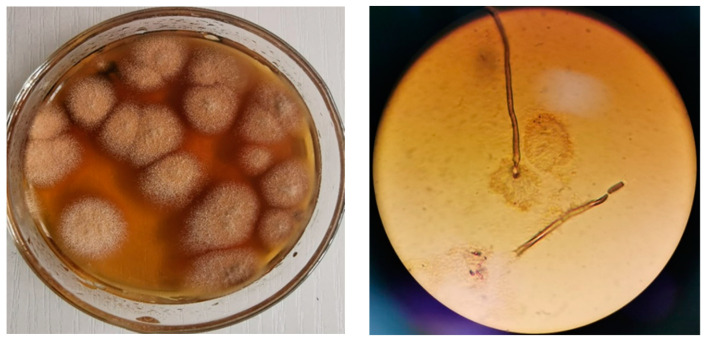
Colonies were grown on a PDA plate (**left**) and morphology (1000×) was observed under a microscope (**right**) of YJ01.

Using gossypol as the sole carbon source, a fungus YJ01 with gossypol biodegrading ability was successfully isolated. On solid medium, colonies of this strain had well-developed mycelium and velvety to flocculent texture and were round, thin, and brown in color with a slightly raised central part and could produce black spores (Figure 2). Podocytes, conidial heads, and conidial stalks were observed under the microscope. The conidial head is columnar, the apical sac is hemispherical, there are two layers of pedicels, the conidium is almost spherical, and the pedicel is short and smooth. The results also showed that YJ01 could be grown on Bengal Red medium, PDA medium, and Czapek Dox medium.

**Figure 3:**
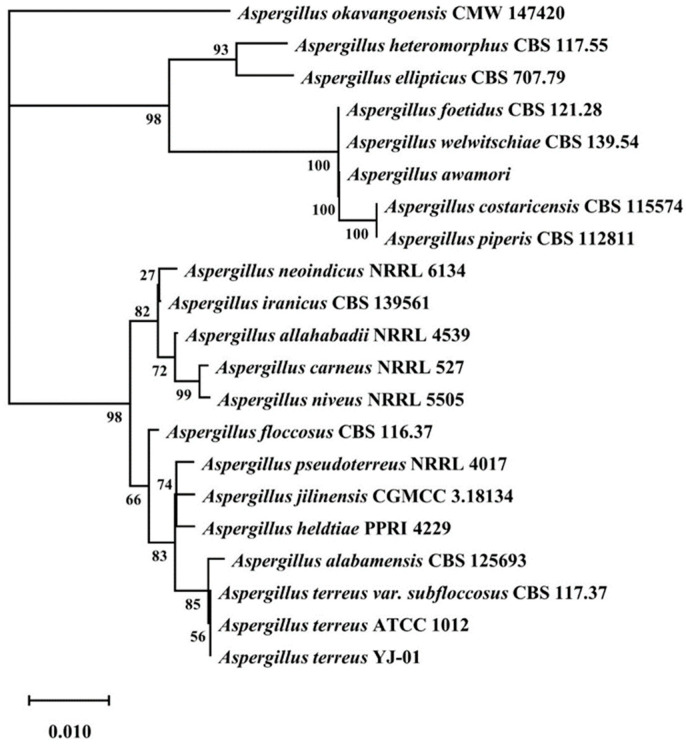
Phylogenetic tree based on ITS sequences of *Aspergillus terreus*-YJ01.

ITS of YJ01 was amplified and sequenced to determine its phylogenetic placement. The analysis results of the ITS sequences showed that YJ01 appeared to be closely related to *Aspergillus terreus* (Figure 3). Based on the morphological and physiological characteristics, as well as the phylogenetic analysis of ITS sequences, we identified YJ01 as *A. terreus*-YJ01.

### 3.2. Biodegradation of Gossypol by YJ01

**Figure 4:**
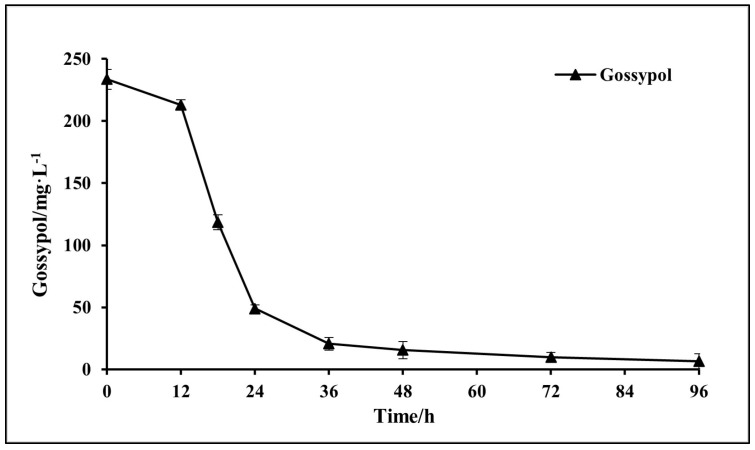
Biodegradation kinetics of gossypol by YJ01.

Initial gossypol of 250 mg·L^−1^ was removed by 97% in 96 h by *A. terreus*-YJ01 (Figure 4), indicating that this isolated fungal strain has a better ability to biodegrade gossypol than any other microorganisms reported [20,29,41]. Different microorganisms have different abilities to biodegrade gossypol [24]. At present, gossypol has been shown to be best biodegraded by fungi such as *Aspergillus niger* and yeast such as *Candida utilis*, *Bacillus subtilis,* and *Rhodococcus erythropolis* [29,44]. Chen et al. obtained a strain of *Aspergillus terreus* capable of degrading gossypol from soil samples from cotton fields and cottonseed oil extraction plants using several rounds of screening, but its biodegradation rate of gossypol reached only 33.6% within 72 h [45]. Compared with the other gossypol-biodegrading microorganisms reported, YJ01 is a promising fungal strain for efficient biodegradation of gossypol.

### 3.3. Biodegradation of Gossypol by Intracellular Crude Enzymes and Extracellular Crude Enzymes

**Figure 5:**
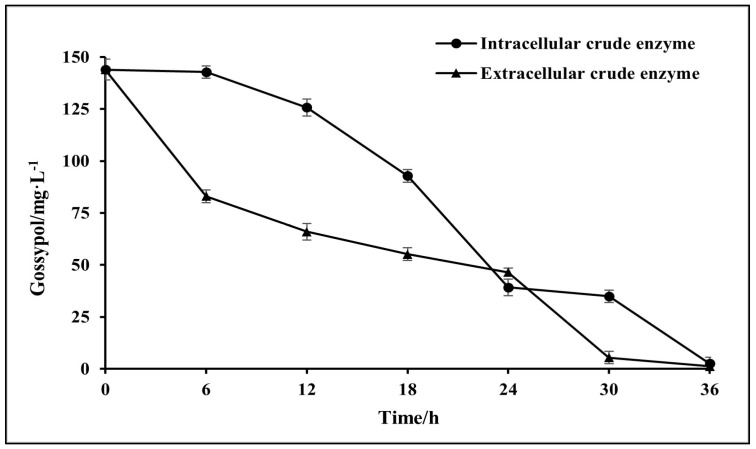
Biodegradation kinetics of gossypol by intracellular crude enzymes and extracellular crude enzymes.

The intracellular crude enzyme of *A. terreus*-YJ01 containing a protein concentration of 2.10 mg·L^−1^ removed 150 mg·L^−1^ of gossypol by 98% within 36 h. Furthermore, 150 mg·L^−1^ of gossypol declined by 99% within 36 h by the extracellular crude enzyme with a protein concentration of 2.13 mg·L^−1^ (Figure 5). Gossypol was removed by both the cells and the intracellular as well as extracellular crude enzymes of *A. terreus*-YJ01 (Figure 4 and Figure 5). Compared with the cells of *A. terreus*-YJ01, its intracellular and extracellular crude enzymes show a higher biodegradation rate, which is significant for the efficient elimination of gossypol from cottonseed meal.

### 3.4. Effects of Medium and Culture Conditions on the Growth and Gossypol Biodegradation of YJ01

**Figure 6:**
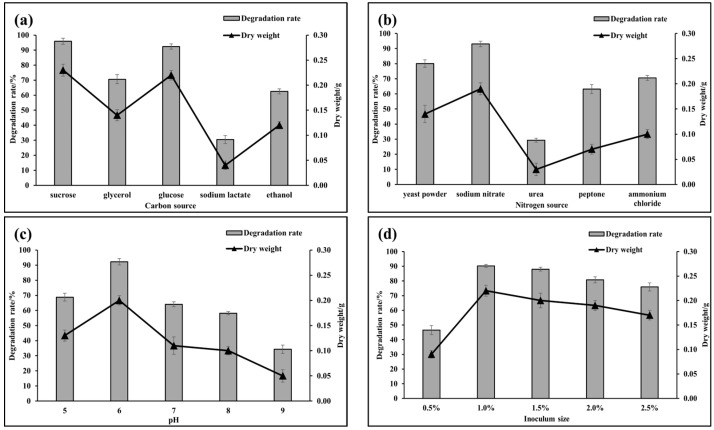
Effects of carbon source (**a**), nitrogen source (**b**), initial pH (**c**), and inoculum size (**d**) of gossypol on growth and gossypol biodegradation ratio by YJ01 cultured for 72 h.

The effects of different carbon sources, nitrogen sources, pH, and inoculum size on the growth of *A. terreus*-YJ01 and biodegradation of gossypol were studied. As shown in Figure 6a, compared with glycerol, glucose, sodium lactate, or ethanol as sole carbon sources, sucrose was found to be an optimal carbon source to support both the growth and gossypol biodegradation of *A. terreus*-YJ01. As shown in Figure 6b, in the medium with yeast powder, sodium nitrate, urea, peptone, and ammonium chloride as sole nitrogen sources, respectively, sodium nitrate was shown to be an optimal nitrogen source to support both the growth of *A. terreus*-YJ01 and gossypol biodegradation. Both the growth of *A. terreus*-YJ01 and the biodegradation of gossypol were promoted at pH 6.0, with a range from 5.0 to 9.0, as shown in Figure 6c. The results also indicated that the inoculum size of 1%, in the range from 0.5% to 2.5%, was an optimal inoculum size for both the growth of *A. terreus*-YJ01 and the biodegradation of gossypol, as shown in Figure 6d.

### 3.5. Genomic Analysis of the Biodegradation of Gossypol by YJ01

To study the mechanism underlying gossypol biodegradation by YJ01, DNA from *A. terreus*-YJ01 samples was extracted, purified, and constructed into a library. The draft genome was sequenced, assembled, and checked using the Illumina Hiseq platform with paired-ends sequencing [46]. This revealed a total length of 31,566,870 bp, with a GC content of 52.27%. The reads were assembled into 275 scaffolds with an N50 of 980,104 bp, and 9737 protein-coding genes, 161 tRNA genes, and 27 rRNA genes were predicted (Table 1).

Table 2 displays the statistics of the second-generation quality control data. Both the original sequence data (18,480,614*2) and the post-quality control data (18,238,868*2) contained a certain number of double-ended reads. In total, 97.45% (Q20) and 93.35% (Q30) of the total bases in the original data were bases with error identification rates less than 0.01 and 0.001, respectively. Following data quality assessments, bases with error identification rates under 0.01 and 0.001 represented, respectively, 97.75% (Q20) and 93.71% (Q30) of the total bases. After quality control, the total base count was 5,537,387,630 bp, compared with the total base count of the original data, which was 5,581,145,428 bp.

**Figure 7:**
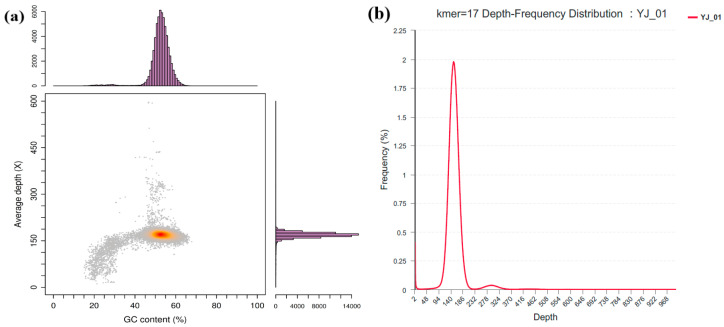
GC_depth distribution (**a**) and K-mer depth-frequency distribution (**b**) analysis results.

Genomic assessments, including GC_depth distribution analysis and K-mer frequency distribution analysis, were performed on the genome sequences generated after assembly to determine whether contamination was present. Figure 7a shows the GC_depth distribution with GC content as the horizontal coordinate and the coverage depth of reads as the vertical coordinate. At the same time, the histograms on both sides reflect the distribution trend in GC content and sequencing depth. From the graph, we can see that most of the spots are distributed in a relatively concentrated area, and the distribution area is narrow, indicating that the sample of the YJ01 genome is of high purity and free from contamination. The horizontal coordinate of the K-mer frequency distribution analysis result graph in Figure 7b represents the sequencing depth, and the vertical coordinate represents the proportion of the frequency to the total frequency at each sequencing depth. The main peak in the graph is relatively complete and has no heterozygous peak or repeat peak and shows a Poisson distribution, indicating that there are few heterozygotes and repeats in the genome and that the quality is good.

**Figure 8:**
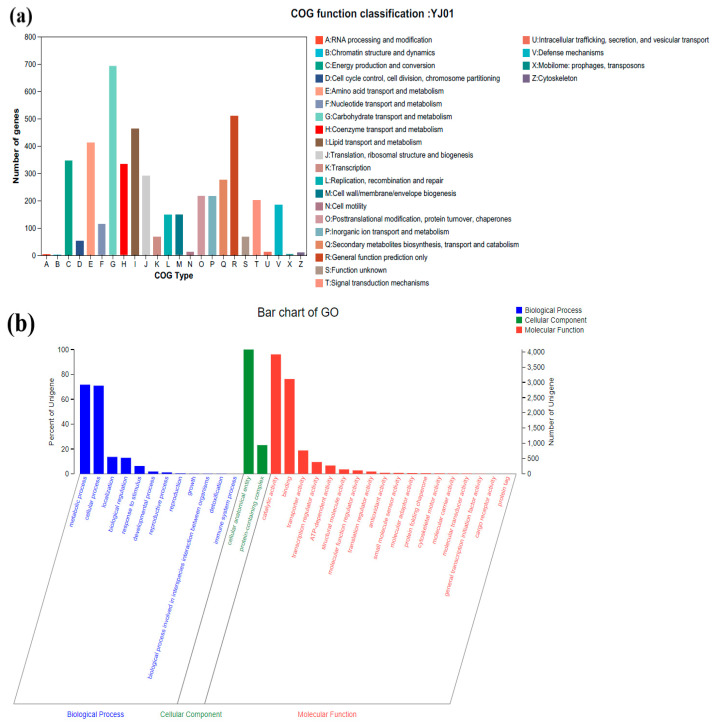

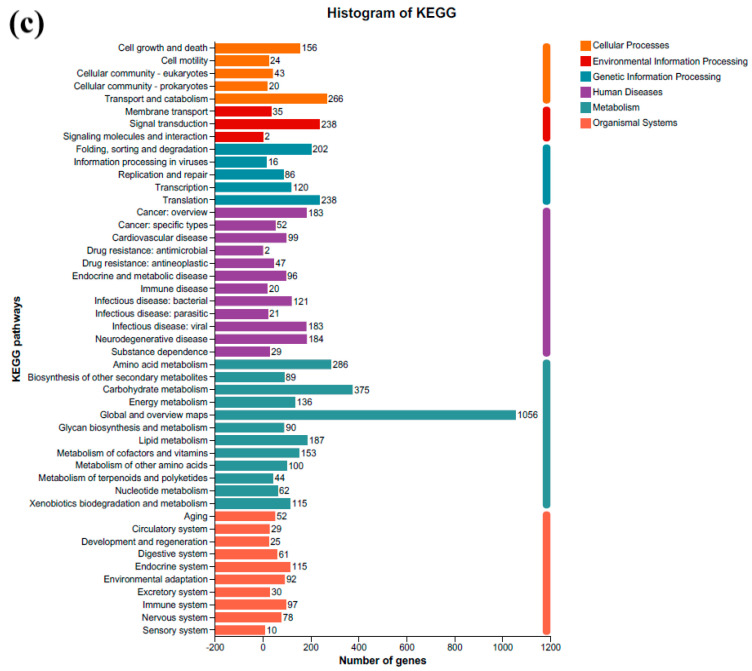
COG (**a**), GO (**b**), KEEG (**c**) annotation classification of YJ01.

The results for COG annotation revealed that 4079 genes (41.89%) in the genome of YJ01 matched the annotation information in the COG database (Figure 8a) and were involved in 25 metabolic pathways. Notably, there were 693 genes related to carbohydrate transport and metabolism (G), which accounted for the highest proportion (17.0%). The number of genes annotated for general function prediction only (R), lipid transport and metabolism (I), amino acid transport and metabolism (E), energy production and conversion (C), and coenzyme transport (H) was 510, 463, 411, 346, and 334, respectively. Furthermore, in GO functional annotation, 7224 coding genes were annotated to biological processes (3866, 53.5%), cellular components (4449, 61.6%), and molecular functions (5778, 80.0%), respectively (Figure 8b). In the biological process, the metabolic process (2923), the cellular process (2890), localization (556), and biological regulation (526) accounted for a large proportion of secondary functions, respectively. The cellular anatomical entity (4075) accounted for the largest proportion of genes annotated in the cellular component. The proportion of genes annotating catalytic activity and binding functions in molecular function was high, with 3916 and 3109 genes, respectively. Additionally, 3514 genes in the KEGG database were matched with annotation information and participated in 47 metabolic pathways in 6 major metabolic hierarchies (Figure 8c). Among them, 1056 genes were associated with the general metabolic pathway, 375 genes with carbohydrate metabolism, 286 genes with amino acid metabolism, and 266 genes with transport and catabolism.

No metabolic pathway map related to gossypol biodegradation was found in KEGG annotations, but according to previous reports [13,29], eight enzymes have been speculated to be directly related to the biodegradation of gossypol by *A. terreus*-YJ01, which were glucose-6-phosphate isomerase, phosphoglycerate kinase, glyceraldehyde-3-phosphate dehydrogenase, malate dehydrogenase, citrate synthase, glutamate dehydrogenase, inorganic pyrophosphatase, and β-1, 4-endo-xylanase (Table 3). Due to the complex structure of gossypol, more energy is required for biodegradation [47]. Glucose-6-phosphate isomerase, phosphoglycerate kinase, and glyceraldehyde-3-phosphate dehydrogenase play important roles in glycolysis to produce energy. In addition, malate dehydrogenase and citrate synthetase produce additional energy through the TCA cycle. Glutamate dehydrogenase and inorganic pyrophosphatase can further regulate body energy. Under the condition of sufficient energy, *Aspergillus terreus* can further participate in the biodegradation of gossypol through β-1, 4-endo-xylanase to achieve gossypol degradation. This provided the biological basis for the identification of functional genes involved in gossypol biodegradation and clarified the biodegradation process.

Gossypol is a derivative of naphthalene, and its biodegradation pathway may be related to the naphthalene metabolic pathway. Naphthalene is an aromatic compound. Its biodegradation process is much more complex than that of glucose and requires more energy [48]. We speculate that a considerable amount of energy is also required to degrade this complex molecule in the process of gossypol biodegradation, so these proteins may play an important role in gossypol biodegradation. In addition, extracellular xylanase and β-1, 4-endo-xylanase have also been identified. A large number of studies have shown that xylanase has some ability to remove crude lignin and can indirectly degrade lignin [26]. It plays an important role in lignin biodegradation. There are phenol hydroxyl groups, alcohol hydroxyl groups, and other active groups in the molecular structure of lignin [25]. Gossypol is one of the polyphenolic hydroxyl groups [27,28]. We speculate that xylanase and β-1, 4-endo-xylanase may be involved in the biodegradation of gossypol through specific pathways.

## 4. Conclusions

*A. terreus*-YJ01, an efficient fungal strain for biodegrading gossypol, was successfully isolated from the soil of cotton stalk piles in Xinjiang Province, China. Sucrose and sodium nitrate proved to be the best carbon and nitrogen sources for the growth of YJ01, and the optimal pH and inoculum size for the growth of YJ01 were 6.0 and 1%, respectively. Under optimal culture conditions, initial gossypol of 250 mg·L^−1^ was removed by 97% within 96 h by YJ01. Moreover, initial gossypol of 150 mg·L^−1^ was also catalyzed by 98% or 99% within 36 h by the intracellular or extracellular crude enzymes of YJ01. These findings clearly demonstrate YJ01’s remarkable capacity for gossypol biodegradation. Eight proteins of interest were found to be associated with the biodegradation of gossypol. Notably, phosphoglycerate kinase and malate dehydrogenase produce energy for the biodegradation process through glycolysis and the TCA cycle, respectively. Glutamate dehydrogenase and inorganic pyrophosphatase can further regulate energy supply. In case of sufficient energy, *Aspergillus terreus* can further participate in the biodegradation of gossypol through xylanase and β-1, 4-endo-xylanase to achieve gossypol biodegradation. The findings of this study provide valuable insights into the metabolism pathway underlying gossypol biodegradation by *A. terreus*-YJ01. The products of gossypol biodegradation by YJ01 are being investigated in order to fully understand the mechanisms underlying gossypol biodegradation.

## Figures and Tables

**Table 1:** Basic information on the genome assembly of YJ01.

Attribute	Value
Sample Name	YJ01
Total Scaffolds No.	275
Total Bases in Scaffolds (bp)	31,566,870
Large Scaffolds No. (>1000 bp)	275
Largest Scaffold Bases (bp)	31,566,870
G+C (%)	52.27
Scaf N50 (bp)	980,104
Scaf N90 (bp)	127,246

**Table 2:** Statistics of the quality control data for second-generation sequencing.

Attribute	Value
Sample Name	YJ01
Insert Size (bp)	500
Read Len (bp)	150
Raw Pair Reads	18,480,614*2
Raw Bases (bp)	5,581,145,428
Raw Q20 (%)	97.45
Raw Q30 (%)	93.35
Clean Pair Reads	18,238,868*2

**Table 3:** Genes and corresponding enzymes related to gossypol biodegradation by YJ01.

Gene ID	Database	Gene Name
gene5756, gene8925	KEGG	xynA
gene0733	KEGG	pgk
gene2649, gene7420	KEGG	-
gene4148	KEGG	ppa
gene2257	KEGG	-
gene1802	KEGG	pgi
gene8994	KEGG	gapA
gene3004, gene7660, gene7979	KEGG	gltA

## Data Availability

Not applicable.
